# New Cap-Holed AlP, GaP, and InP Nanotubes

**DOI:** 10.1021/acsomega.3c08486

**Published:** 2024-01-02

**Authors:** Raúl Mendoza-Báez, Dolores García-Toral, Juan Francisco Rivas-Silva, Akari Narayama Sosa Camposeco, Sandra Esteban Gómez, Gregorio Hernández Cocoletzi, Antonio Flores-Riveros

**Affiliations:** † Departamento de Química, Centro de Investigación y de Estudios Avanzados del IPN (Cinvestav), Av. IPN 2508, Col. San Pedro Zacatenco, México City 07360, México; ‡ Facultad de Ingeniería Química, Benemérita Universidad Autónoma de Puebla, Av. San Claudio y 18 Sur S/N, San Manuel, Puebla 72570, México; § Instituto de Física, Meritorious Autonomous University of Puebla, 72570Puebla,Mexico; ∥ Instituto de Física, Benemérita Universidad Autónoma de Puebla, Av. San Claudio y Blvd. 18 Sur, Col. San Manuel, Puebla 72570, México

## Abstract

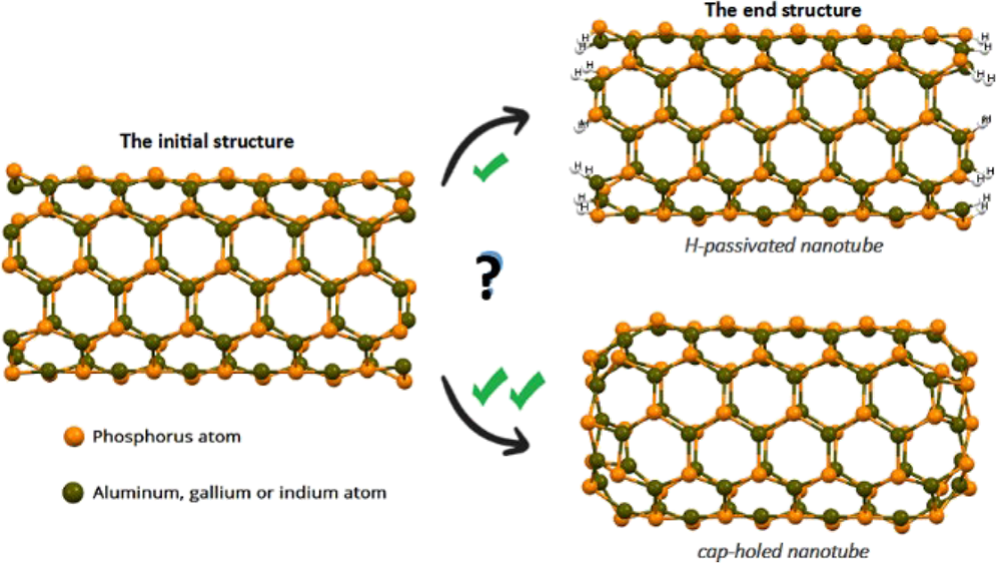

The structural, vibrational, and electronic properties of new inorganic X-phosphide nanotubes (*ch*-XPNT), with X = Al, Ga, or In and chirality of (5,5), are investigated. These new NTs display *cap-hole* ends, with the *cap-hole* features induced by the nonpassivated ends. Studies are based on density functional theory (DFT) using the M06-2X, PBE, and B3LYP functionals together with the LanL2DZ basis set. All nanostructures have been relaxed by minimizing the total energy, assuming a nonmagnetic nature and a total neutral charge. Note that the *cap-hole* NTs are terminated by a 10-atom ring, which in turn favors the geometrical ordering and yields stable structures. The (5,5) *ch*-XPNT are highly electrophilic and nonpolar, in addition to having high solvation energy values. Let us remark that solvation energies are produced by the intermolecular forces that involve the induced dipoles. Structural and vibrational results show that the X–P bonds are single bonds. Finally, results suggest that the inorganic nanotubes are structurally stable with semiconductor features, which means that their functionalization may yield interesting future applications.

## 1 Introduction

The interesting structural, physical, chemical, mechanical, optical, electronic, and magnetic properties of carbon nanotubes (CNTs), which were discovered by S. Iijima,^1^ have motivated studies and design of new inorganic nanotubes with similar atomic configurations as those of CNTs. These inorganic NTs may be formed by the III–V elements of the periodic table. At this point, mention must be made on the existence of notable nanotubes such as gallium phosphide (GaPNT), aluminum phosphide (AlPNT), and indium phosphide (InPNT) nanotubes, which are inorganic and indeed quite interesting because of their properties for practical applications. GaPNTs have been synthesizes by Wu et al.^2^ for the first time in 2005. The structural, electronic, magnetic, and reactivity properties of GaPNTs in the armchair and zigzag chirality have been investigated by computational chemistry. Results indicate varied and interesting applications such as drug delivery, particularly for 5-fluorouracil, hydrogen storage, as well as in the fabrication of spintronic and nanoelectronic devices.^3−10^ AlPNTs have been studied and characterized computationally for the first time by Lisenkov et al.^11^ The use of these NTs as hydrazine sensors for biomedical applications and as sensors of toxic gases such as acrolein and hydrogen sulfide has been investigated.^12−14^ On the other hand, the inclusion of transition metals (Co, Ni, Mn) in the structure of AlPNTs allows modulating their electronic and magnetic properties.^15^ Another example of NTs is that concerned with the InPNTs. These NTs have been synthesized by applying the ablation laser technique by Bakkers and Verheijen in 2003.^16^ Experimental and theoretical studies of the InPNTs' structural and electronic properties have been reported.^17,18^ Recently, the (4,0) InPNTs have been proposed to transport the anticancer drug cisplatin.^19^ Sakharova et al.^20^ have reported numerical calculations of the elastic properties corresponding to all three inorganic nanotubes: AlP, GaP, and InP.

Most of the nanotubes mentioned above are open-end and passivated structures; that is, the atoms at every end of the nanotube contain no dangling bonds, as they are saturated with hydrogen atoms. However, this form of termination is merely a resource used for the finite theoretical–molecular study of these nanotubes because it has not been reported experimentally. The main nanotube endings that have been observed experimentally are closed caps with graphitic layers, caps coated with metallic particles or clusters, free caps with dangling atoms (highly reactive), and flat-cap nanotubes.^21−34^ However, these findings are limited almost solely to CNTs (mainly) and BNNTs. This suggests that AlPNTs, GaPNTs, and InPNTs may also have a closed-cap type termination. The main motivation is to provide a specific theoretical study on the structural conformation of the caps in these inorganic nanotubes and their influence on the electronic properties because, as far as we know, these NTs have not been studied yet. Results show that the formation of a new type of termination induced by nonpassivation is favored, promoting a restructuring given by the formation of four- and six-member rings.

On the basis of the DFT calculations, studies are performed of the structural, vibrational, and electronic properties of aluminum, gallium, and indium phosphide armchair NTs with (5,5) chirality and with cap-hole ends: (5,5) ch-XPTN (where X = Al, Ga, or In). The chemical stability and reactivity are explored using the cohesive energy (*E*_coh_), dipole moment (μ →), solvation energy (Δ*E*_solv_), and global molecular descriptors such as the chemical potential (μ), global hardness (η), and the electrophilicity index (ω^±^). The dependence of these properties on the nature of the p-block metal is emphasized. The results show that these inorganic nanotubes are structurally stable with semiconductor characteristics, suggesting their use in the manufacture of nanoelectronic devices. Moreover, these ch-XPNTs (X = Al, Ga, In) can be extended to other chiralities and serve as a new representation or molecular model for their quantum–chemical study in interactions with, for example, organic molecules (drug delivery, gas sensor, adsorption of contaminants, among others).

## 2 Theoretical Methods

Studies are performed on the structural, vibrational, and electronic properties of the AlP, GaP, and InP nanotubes in the armchair geometry with *cap-hole* ends, namely, (5,5) *ch*-AlPNTs, (5,5) *ch*-GaPNTs, and (5,5) *ch*-InPNTs, respectively. First-principle calculations, using the density functional theory (DFT), have been done to investigate the NTs. Each nanotube is composed of 120 atoms: 60 atoms of P and 60 atoms of Al, Ga, or In. The structural relaxation, total energy, dipole moment, and energy of the orbitals HOMO and LUMO have been determined by applying the functionals M06-2X,^35^ PBE,^36^ and B3LYP^37−39^ together with the LanL2DZ basis set, as implemented in the Gaussian code.^40^ LanL2DZ is a basis set that contains the effective core potential (ECP) representations of the electrons for the atoms beyond the third-row elements.^41−44^ The stabilized NTs are of singlet multiplicity (M = 1) with neutral charge. The vibrational frequency calculations were performed to corroborate the structural stability. Water as a solvent has been considered according to the conductor-like polarizable continuum model (CPCM)^45−47^ to determine the solvation energy of NTs. The system stability and reactivity have been determined through the molecular quantum descriptors such as the chemical potential (μ), global hardness (η), and electrophilicity index (ω), which are obtained from the HOMO and LUMO energy values, according to the Koopmans theorem:^48^
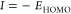
1
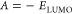
2where *I* is the ionization potential and *A* is the electronic affinity. Therefore, the chemical potential (μ) and global hardness (η) may be expressed as follows:
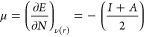
3
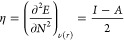
4Here, *E* is the total energy, *N* is number of electrons, and ν(*r*) is the external potential of the system.^49^ The global electrophilicity index, as described by Parr et al.,^50^ is a relationship between the electronic chemical potential and the global hardness (ω = μ^2^/2η). This descriptor represents the system stabilization energy when it is saturated by the surrounding electrons. In this work, distinction is made between the electron-acceptor (ω^+^) and the electron-donor (ω^–^) power, which are calculated according to the following equations:^51,52^
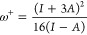
5
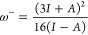
6

High ω^+^ values indicate that the system exhibits a large capacity to accept charge; in contrast, small ω^+^ values is characteristic of electron-donor systems. Moreover, the electron-donor (ω^–^) value allows calculating the nucleophilicity index (*N*″) according to the Roy et al.^53−55^ mathematic expression:
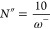
7The system stability may be determined using the cohesive energy (*E*_coh_) as defined below. This is the needed energy to break all bonds, leaving the system with the individual components.^56−60^
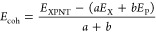
8where *E*_XPNT_ is the total energy of the nanotubes; *E*_X_ and *E*_P_ are the X (group III atom) free atom total and phosphorus free atom total energy, respectively; and *a* and *b* stand for the number of X and phosphorus atoms in the NT, respectively. *E*_coh_ is expressed in eV/atom; a stable geometry exhibits negative cohesive energy.^56−60^

The NT solvation energy (Δ*E*_solv_) has been determined using the total energy of the optimized configuration in a vacuum (*E*_XPNT_) and in water (*E*_XPNT_^CPCM^) as shown in the following equation:
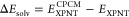
9In this equation, negative values are for systems with a high degree of solubility.

## 3 Results and Discussion

### 3.1 Structural Properties

**Figure 1:**
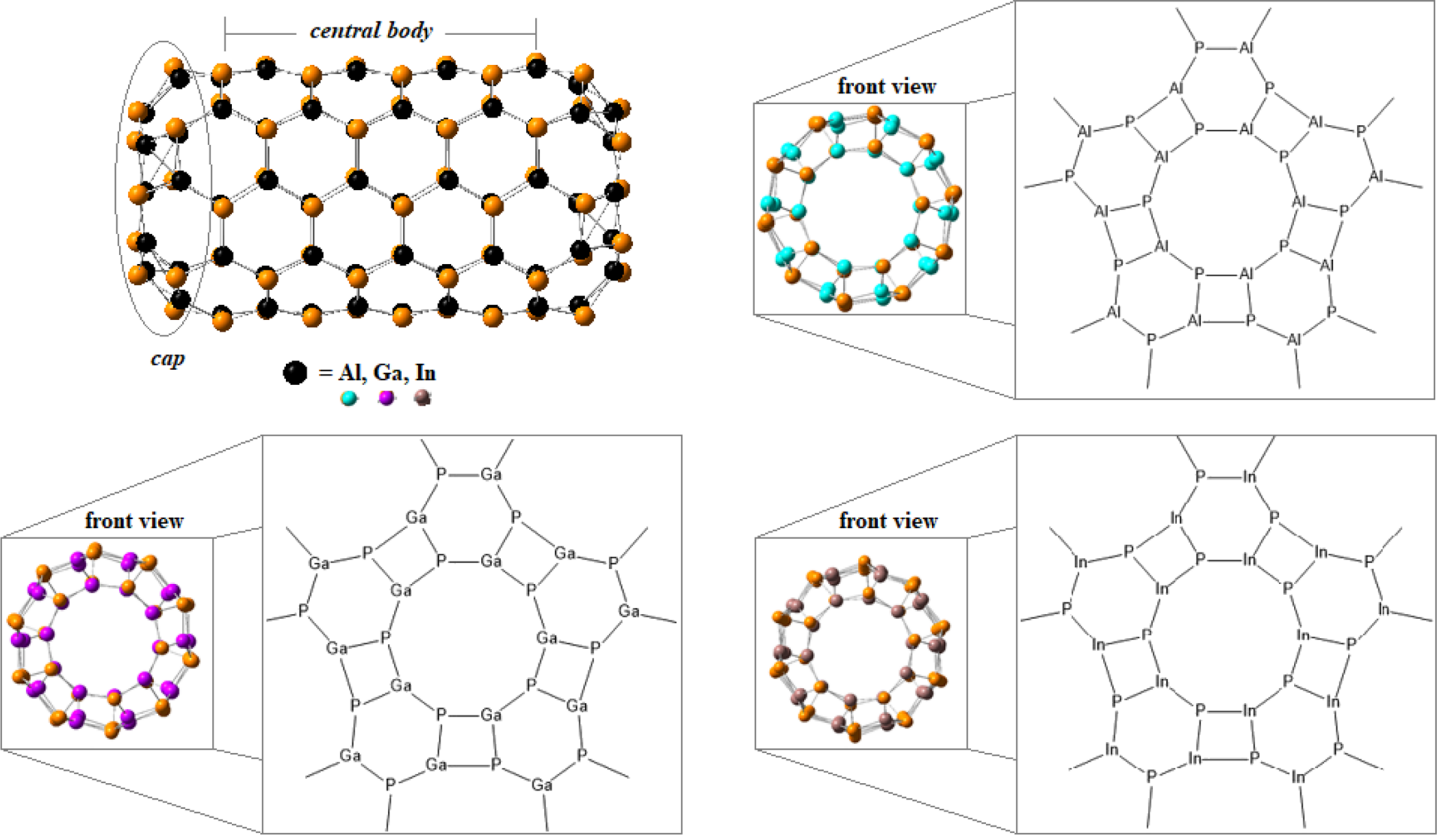
Lateral and front view of the (5,5) *ch*-XPNT in the relaxed geometry: *cap* and body. Zoom-in: front view of the nanotube cap-hole (10-atom ring). Atom colors: orange, phosphorus; cyan, aluminum; purple, gallium; and brown, indium.

**Figure 2:**
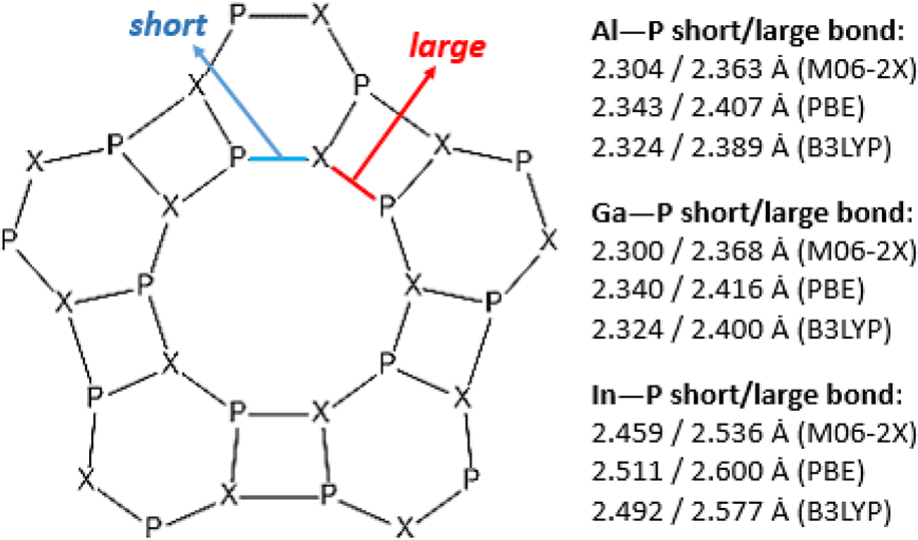
Large and short bond lengths of the 10-atom ring.

Table 1 summarizes the X–P bond lengths (with X = Al, Ga, In), bond angles, *cap* diameters, and axial length of all relaxed structures. It is noted that there is no significant difference in the values obtained based on the different functionals used, except for the X–P–X angle, where the PBE functional showed the smallest value for the three structures. The P–X–P angles are larger than those of the X–P–X structures. These differences may be explained in terms of the annular distribution of the *cap* ends as they form a hexagonal X_3_P_3_ geometry. Note that the cap ends close the NTs, which in turn induce the bend in the structure and modify the bond lengths (P–X) and bond angles (P(X)–X(P)–P(X)) as compared with those in the NT central part (Table 2). Figure 1 displays a lateral view and a front view of the NTs. The “*cap-hole*” presence with a 10-atom ring is noted, with each ring formed with adjacent cycles of four and six members in an alternated fashion. The diameters have been determined using this decagon. In all cases, the X–P bond lengths exhibit the following tendency: 4-ring >6-ring >10-ring > central section (Table 2). These 10-atom rings display X–P bond lengths short and large in an alternating form. The six-atom rings show the shortest bond lengths, whereas the four-atom rings show the largest bond length (Figure 2). Previous works have reported that inorganic clusters of BN and Al_2_O_3_, which have alternating four- and six-membered rings, are the most stable arrangements.^28,61^ The formation of a *cap-hole* geometry induces stable restructured NTs; that is, intramolecular passivation exists to yield stability. Zhu et al.^62^ have pointed out that the BNNTs containing four- and eight-atom rings have small energy gaps (semiconductors) as compared to those BNNTs with six-atom rings, which are isolators.

**Table 1:** Nanotubes' Relaxed Structural Parameters: (5,5) ch-XPNT (X = Al, Ga, In)

**nanotube**	lengths (Ȧ)			**angle (°)**	
**(5,5)***ch***-AlPNT**	**diameter**	**axial axis**	X–P bond	X–P–X	P–X–P
M06-2X	7.4625	21.0659	2.3339	116.90	120.70
PBE	7.4993	21.1010	2.3692	113.01	120.39
B3LYP	7.4986	21.1313	2.3515	115.37	120.76
**(5,5)***ch***-GaPNT**					
M06-2X	7.4154	20.8731	2.3304	114.68	120.70
PBE	7.4686	20.9363	2.3722	110.62	119.62
B3LYP	7.4676	20.9702	2.3544	112.85	120.34
**(5,5)** *ch***-InPNT**					
M06-2X	7.9542	22.3059	2.4973	114.37	120.80
PBE	8.0148	22.4218	2.5521	109.62	119.26
B3LYP	8.0119	22.4726	2.5294	112.26	120.19

**Table 2:** X–P Bond Lengths at Different Regions of the NTs (Body, 4-Ring, 6-Ring, 10-Ring) of the (5,5) *ch*-XPNTs (X = Al, Ga, In)

X–P bond	(5,5) *ch*-AlPNT			(5,5) *ch*-GaPNT			(5,5) *ch*-InPNT		
*functional*	**M06-2X**	**PBE**	**B3LYP**	**M06-2X**	**PBE**	**B3LYP**	**M06-2X**	**PBE**	**B3LYP**
body	2.2836	2.3129	2.2961	2.2727	2.3207	2.3145	2.4301	2.4973	2.4691
4-ring	2.3739	2.4124	2.3944	2.3758	2.4149	2.3833	2.5486	2.5986	2.5780
6-ring	2.3442	2.3822	2.3641	2.3427	2.3810	2.3654	2.5132	2.5603	2.5411
10-ring	2.3335	2.3750	2.3565	2.3340	2.3780	2.3620	2.4975	2.5555	2.5345

Previous works report that the bond length for Ga–P single bonds is between 2.31 and 2.35 Ȧ, depending on the system characteristics.^63,64^ Therefore, the Ga–P bond nature in the (5,5) *ch*-GaPNT corresponds to a single bond; however, this is modulated by the geometric distortion. These Ga–P bond length values are similar to those reported for the open GaPNTs^8,10^ with chirality (5,5) and (10,0). The (5,5) *ch*-GaPNTs' diameters are quite similar for both NT ends, which indicate that no buckling effect is manifested, in contrast to those open GaPNTs investigated by Kamal et al.^10^ (periodic NTs). Regarding the Al–P bond length, reports indicate that the single bonds lie between 2.330 and 2.681 Ȧ, depending upon the interacting species.^65,66^ This suggests that the Al–P bond in (5,5) *ch*-AlPNT corresponds to a single bond (Tables 1 and 2). The Al–P bond lengths are similar to those of the zigzag AlPNTs reported in the literature.^11,14,15,67,68^ Finally, the In–P bond length is of the order of 2.49–2.55 Ȧ, which are values similar to 2.541 Ȧ, as reported for the pure InP structure.^69,70^ Douglas and Theopold^71^ reported an experimental value of 2.62 Ȧ corresponding to the In–P single bond in an In_2_P_2_^71^ cluster with square geometry. Ming-Der Su et al.^72^ have suggested a triple In–P bond with lengths between 2.3 and 2.46 Ȧ as determined computationally. It may be stated that the In–P bonds in the (5,5) *ch*-InPNT display single bond characteristics. We have also confirmed the thermal stability by performing ab initio molecular dynamics simulation (CASTEP) using a canonical ensemble, where, after heating at 300 K, it is found that the potential energy fluctuates around a constant magnitude of energy and there is no structural distortion at the end of the simulation. With the GGA-PBE, ultrasoft pseudopotentials, for 1 ps, step of 0.001 ps with 1000 iterations, Nose–Hoover at room temperature.

### 3.2 Vibrational Analysis

**Figure 3:**
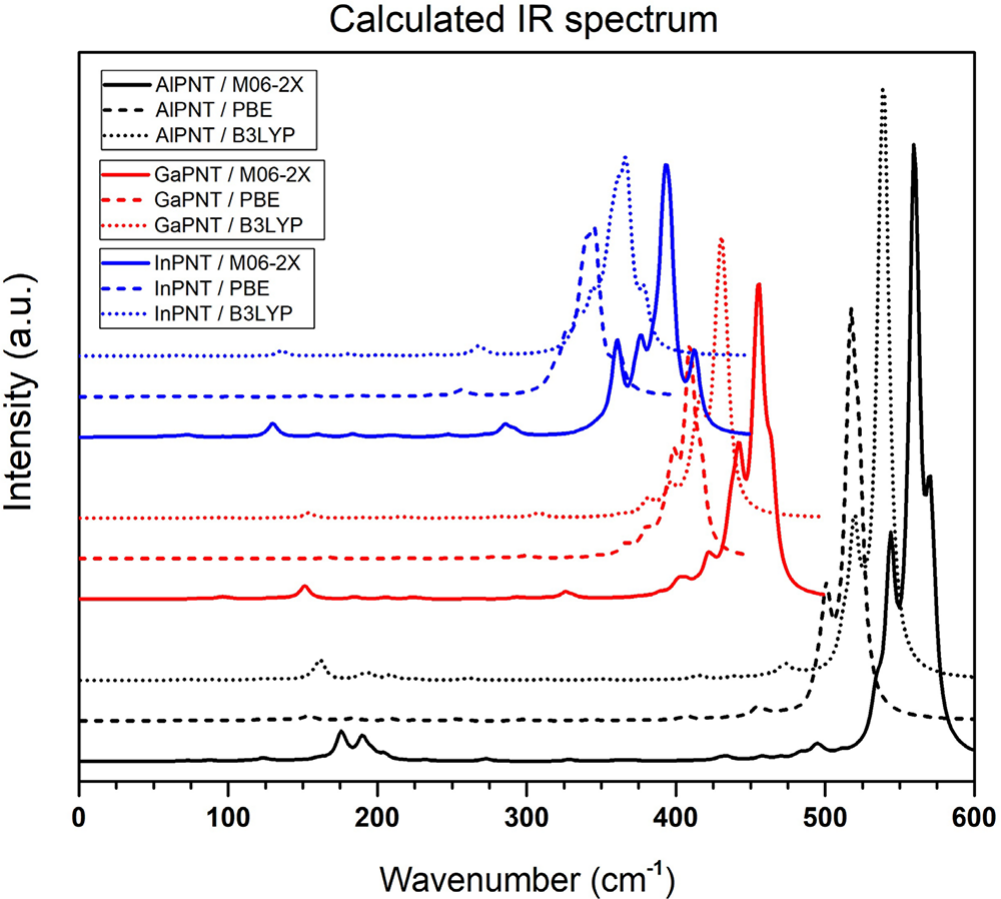
Calculated IR spectra for the (5,5) *ch*-AlPNTs (black curves), (5,5) *ch*-GaPNTs (red curves), and (5,5) *ch*-InPNTs (blue curves). Calculations were done using different functionals: M06-2X (solid curves), PBE (dashed curves), and B3LYP (dotted curves).

The calculated IR spectra have demonstrated that all NTs display nonimaginary frequency values, which indicate that the structures reached the global minima. Figure 3 displays the calculated infrared spectra of the three different nanotubes. The wavenumber ranges from 0 to 600 cm^–1^; the solid, dashed, and dotted curves correspond to results obtained using the M06-2X, PBE, and B3LYP functionals, respectively. Note that the shape, width, and intensity are quite similar regardless of the functional; however, the spectra are wavenumber dependent (cm^–1^). Results obtained with the M06-2X functional are shifted to larger wave numbers; these are followed by those calculated with the B3LYP functional. Spectra calculated with the PBE functionals exhibit the smallest wavenumbers. Table 3 summarizes the results, where vibrational modes are described and assigned to the most representative peaks. The vibrational modes are labeled taking into account the more representative peaks, in accordance with Figure 3, and remarking the differences induced by the functionals. In all three cases, the highest intensity peaks are due to the central body stretching. It is noted that for the same vibrational mode, the wavenumber value (ν∼ in cm^–1^) is ν ∼ _In–P_ < ν ∼_Ga–P_ < ν ∼_Al–P_. This means that the force constant of the In–P bond is the smallest, meaning that this is the chemical bond with the smallest force of all three (X–P, X = Al, Ga, In).

**Table 3:** Calculated Vibrational Frequencies (in cm^–1^) of the Principal Vibrational Modes Corresponding to the NTs: (5,5) *ch*-XPNTs (X = Al, Ga, In)

	**M06-2X**			**PBE**			**B3LYP**		
**vibrational Mode**	**(5,5)***ch***-AlPNT**	**(5,5)***ch***-GaPNT**	**(5,5)***ch***-InPNT**	**(5,5)***ch***-AlPNT**	**(5,5)***ch***-GaPNT**	**(5,5)***ch***-InPNT**	**(5,5)***ch***-AlPNT**	**(5,5)***ch***-GaPNT**	**(5,5)***ch***-InPNT**
*bending*: central body	175.71	151.39	129.86	154.12	166.46	155.53	161.83	154.14	135.73
*bending*: caps	189.91	184.87	183.34	209.47	215.49	185.72	208.34	215.88	181.02
*stretching*: caps	494.32	325.79	291.85	455.07	298.91	255.15	472.46	281.16	266.97
*stretching*: full nanotube	543.82	442.3	376.48	500.72	380.09	326.15	519.43	396.13	334.11
*stretching*: central body^a^	560.16	456.07	395.67	516.89	409.81	346.40	538.62	430.38	367.42
*stretching*: 10-ring	570.51	463.73	412.64	522.72	416.84	361.32	540.51	432.92	378.84

a Vibrational mode with the largest intensity (a.u.)

**Figure 4:**
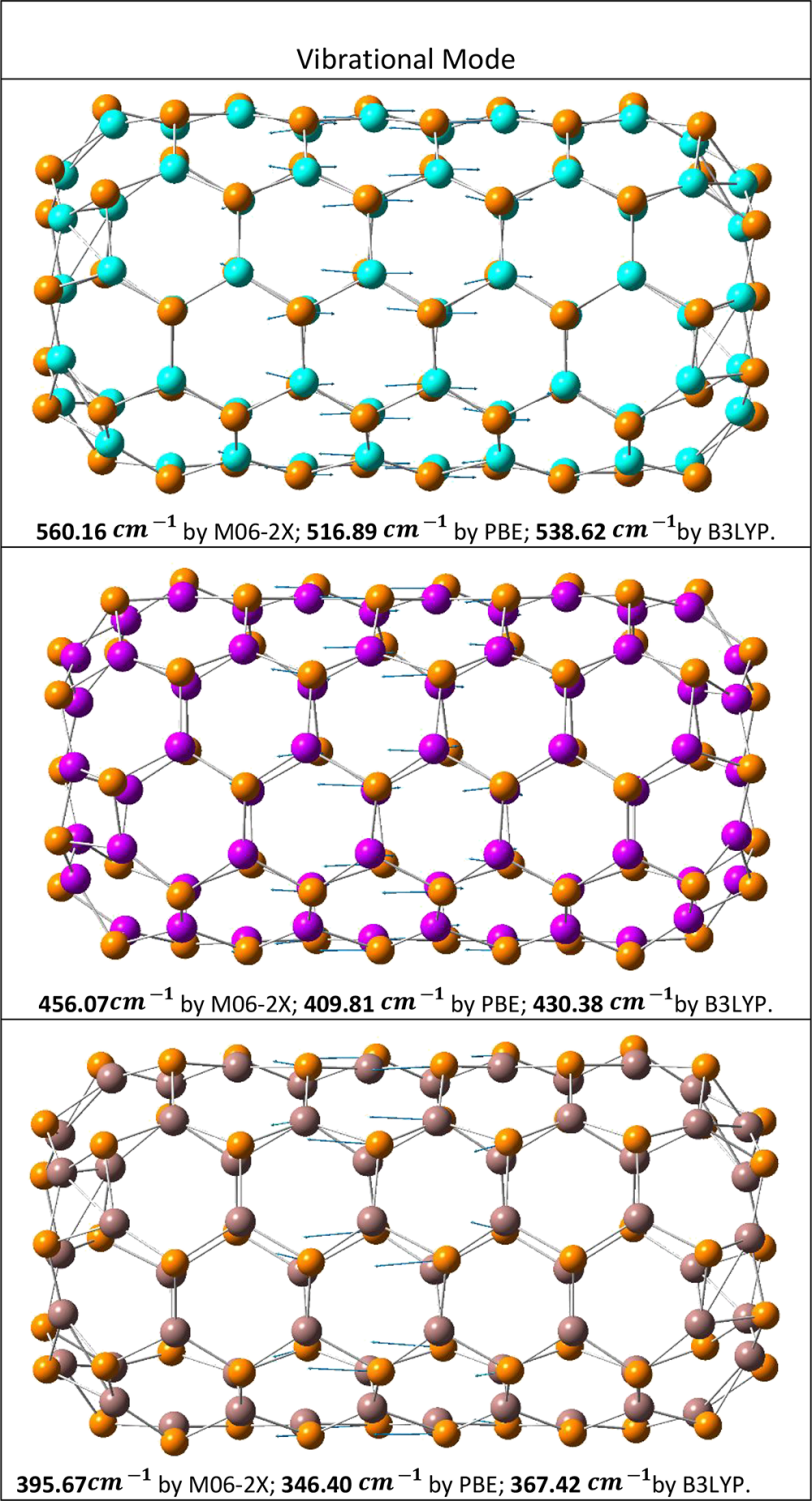
Illustration of the vibrational frequencies for the central body corresponding to the highest intensity vibrational mode: (5,5) *ch*-AlPNTs, (5,5) *ch*-GaPNTs, and (5,5) *ch*-InPNTs. Calculations were performed by using different functionals: M06-2X, PBE, and B3LYP. Atom colors: orange, phosphorus; cyan, aluminum; purple, gallium; and brown, indium.

It is found that adsorption may occur in the optical range (Figure 4). Moreover, it is found that the modes manifest themselves along the nanotube axis; that is, they belong to the longitudinal optical mode.

### 3.3 Electronic Properties

#### 3.3.1 HOMO and LUMO Orbitals

**Figure 5:**
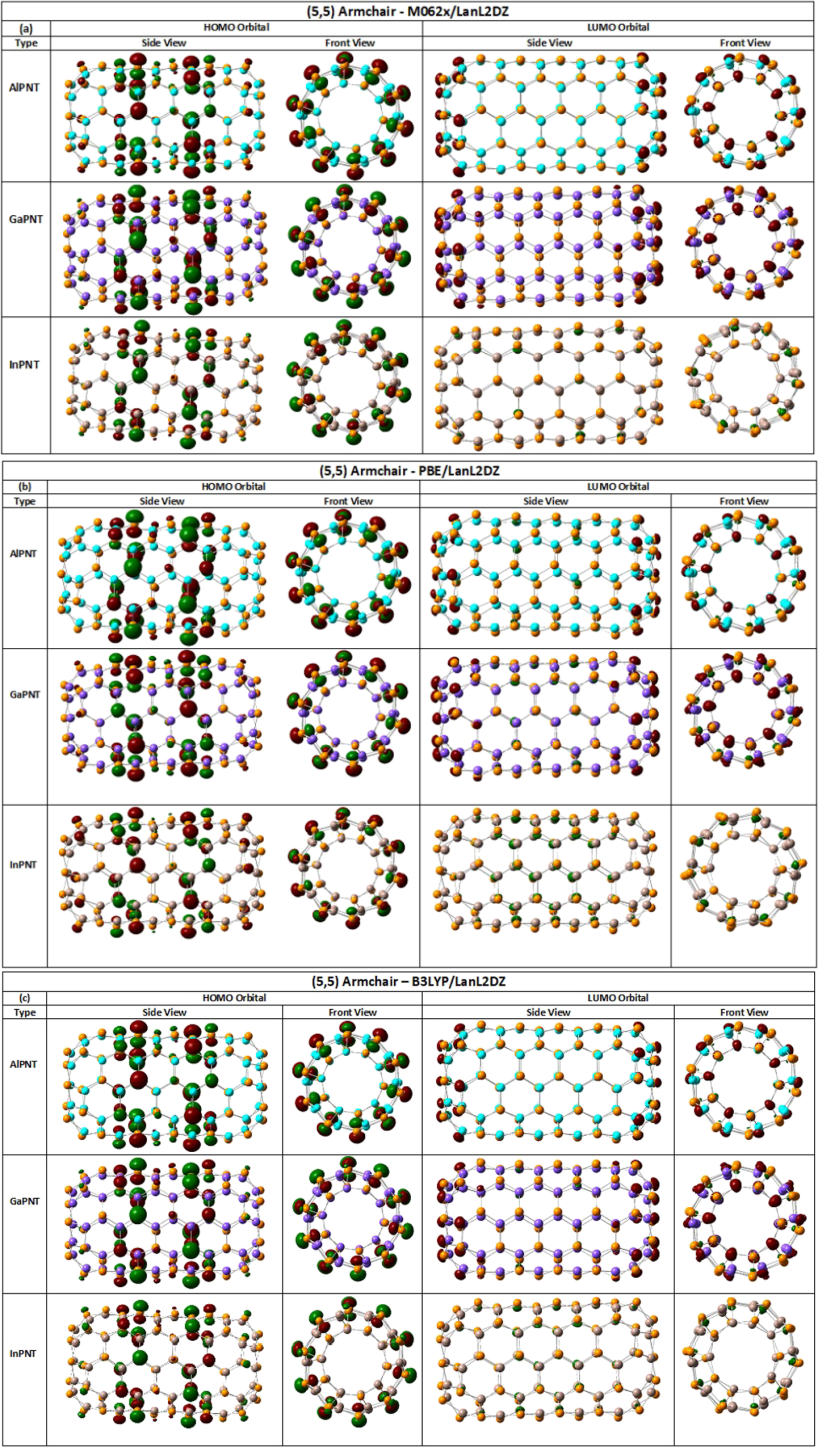
Spatial distribution of the HOMO and LUMO orbitals through the (5,5) armchair-type AlPNT, GaPNT, and InPNT, without passivation. Atom colors: orange, phosphorus; cyan, aluminum; purple, gallium; and brown, indium. Calculated (a) DFT/M06-2X/LanL2DZ, (b) DFT/PBE/LanL2DZ, and (c) DFT/B3LYP/LanL2DZ.

The molecular orbital HOMO and LUMO distributions are shown in Figure 5. In all cases, most of the HOMO orbitals' density is distributed at the nanotube body central zone, mainly on the phosphorus atoms, which are the more electronegative. On the other hand, the LUMO orbitals are mainly distributed on the nanotube caps; however, the (5,5) *ch*-InPNT displays these orbitals within the NT. It is worth mentioning that these distributions are independent of any functional invoked in this work. The nanotubes' stability and reactivity are determined with the aid of the global molecular descriptors, such as the chemical potential (μ), global hardness (η), electrophilicity (ω) index and nucleophilicity (*N*″), as well as the HOMO–LUMO energy gap (*E*_g_) values. Table 4 summarizes these quantities.

**Table 4:** HOMO and LUMO Orbitals' Energies, Molecular Gap (HOMO–LUMO), Ionization Potential (*I*), Electronic Affinity (*A*), and Global Molecular Descriptors (η, μ, ω^±^, *N*″)^a^

nanotube	**(5,5)***cp***-AlPNT**			**(5,5)***cp***-GaPNT**			**(5,5)***cp***-InPNT**		
**descriptors**	**M06-2X**	**PBE**	**B3LYP**	**M06-2X**	**PBE**	**B3LYP**	**M06-2X**	**PBE**	**B3LYP**
EHOMO	–7.4097	–5.9647	–6.3920	–7.5708	–6.0981	–6.5607	–7.3239	–5.8951	–6.3057
ELUMO	–2.8762	–4.2287	–3.6055	–3.2401	–4.4028	–3.9239	–3.5040	–4.5761	–4.1805
*E*_g_ (|H–L| gap)^b^	4.5334	1.7361	2.7864	4.3307	1.6953	2.6368	3.8199	1.3189	2.1252
*I* = −EHOMO	7.4097	5.9647	6.3920	7.5708	6.0981	6.5607	7.3239	5.8951	6.3057
*A* = −ELUMO	2.8762	4.2287	3.6055	3.2401	4.4028	3.9239	3.5040	4.5761	4.1805
μ = −(*I + A*)/2	–5.1430	–5.0967	–4.9987	–5.4054	–5.2504	–5.2423	–5.4140	–5.2356	–5.2431
ω(+)	3.5463	12.5227	6.6422	4.3148	13.7419	7.9660	5.2050	18.2477	10.4465
ω(−)	8.6893	17.6194	11.641	9.7202	18.9924	13.2083	10.6189	23.4833	15.6896
*N*″	1.1508	0.5676	0.8590	1.0288	0.5265	0.7571	0.9417	0.4258	0.6374

a All parameters are given eV unities.

b Half the energy gap (*E*_g_) value is equal to the global hardness (η); seeeq 10*.*

It should be mentioned that the molecular *E*_g_ value is functional dependent. The *E*_g_ values obtained using the M06-2X/LanL2DZ functionals are the largest followed by those calculated with B3LYP/LanL2DZ. Finally, the PBE/LanL2DZ functionals yield the smallest values (Figure S1).

The *E*_g_ value of the (5,5) *ch*-GaPNT is 2.63 eV and that of the (5,5) *ch*-AlPNT is 2.78 eV, as obtained using the B3LYP/LanL2DZ. Experimental *E*_g_ values of GaP and AlP in the bulk have been reported as 2.32 and 2.52 eV, respectively.^10,15,73,74^ Moreover, the *E*_g_ of the (5,5) *ch*-InPNT is 2.12 eV, as calculated with the B3LYP/LanL2DZ, which is similar to that experimental value of ∼1.75 eV reported^17,75^ for the InP NT. Nevertheless, it is currently not an easy task to determine the appropriate method to accurately evaluate the *E*_g_ value because of the lack of experimental data and theoretical calculations for the systems at hand. The B3LYP functional has yielded good results for the HOMO and LUMO levels (to determine *E*_g_) of huge molecules and polymers,^76,77^ being these molecules electrophosphorescent host. Mention shall be done that Takao Tsuneda has investigated silver clusters with results indicating that the PBE/LanL2DZ method underestimates the HOMO energy these in turn yield an underestimated HOMO–LUMO^78^ energy gap. These results agree well with those reported in Table 4. On the other hand, it has been reported that the HOMO–LUMO energy gap of *C*_60_ and *C*_70_ is overestimated by the M06-2X functionals as compared with the experimental data; however, the B3LYP functional yields better results.^79^ As a general trend, the energy *E*_g_ experimental data show a small decrease with the increase in the atomic number of metals corresponding to the *p* block, that is, *E*_gAlPNT_ > *E*_gGaPNT_ > *E*_gInPNT_.

On the basis of the results reported here using the DFT/B3LYP/LanL2DZ methods, the (5,5) *ch*-XPNT (X = Al, Ga, In) nanotubes may be proposed as semiconductors (2.1 < *E*_g_ < 2.8 eV).

#### 3.3.2 Global Molecular Descriptors and the Cohesive Energy *E*_coh_

The global molecular descriptors are widely invoked to theoretically study the stability and reactivity of different molecular systems, among them are the III–V nanotubes.^80−83^

Figure S2 (see Supporting Information) displays the μ and *E*_g_ behavior of each (5,5) *ch*-XPNT. Recall that the chemical potential measures the electron capability of leaving a system in equilibrium. Therefore, a system that can retain electrons exhibits a more negative μ.^84,85^ The (5,5) *ch*-InPNT system has μ with the largest absolute value (more negative); nevertheless, this value is quite similar to that of the (5,5) *ch*-GaPNT structure. The chemical potential descriptor (μ) displays only small variations with respect to the functionals used in the calculations. The global hardness (η) accounts for the system resistance to the charge transference. Therefore, a better stability will have high η values. It is noted that the η values decrease as the group III elements go from Al to In; a similar trend is observed in the molecular gap *E*_g_. This is because these two parameters are directly proportional, as shown in the following equation:
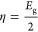
10Concerning the systems' electrophilicity ω^+^ values, these indicate that the (5,5) *ch*-InPNT structures have a better capacity of charge acceptation, that is, the largest electron-acceptor power (). According to eq 5, it is apparent that ω^+^ shows good electronic affinity (*A*), which in turn is determined by the LUMO energy (*A* = −*E*_LUMO_). Therefore, it is reasonable to have (5,5) *ch*-InPNT structures with the LUMO as the level with the smaller energy; in other words, these NTs are the most electrophilic. Nevertheless, all the XPNTs investigated here may be considered as electrophilic NTs provided that ω > 1.5 eV, in agreement with the scale established by Domingo et al.^53,86^ for organic molecules. It should be mentioned that no scale is available for inorganic systems. Moreover, the nucleophilicity (*N*″) is smaller for the (5,5) *ch*-InPNT; in contrast, the (5,5) *ch*-AlPNT structures exhibit greater *N*″ values. However, all structures show an electrophilic character.

The cohesive energy (*E*_coh_) values are summarized in Table 5. All *E*_coh_ values are negative, which indicate the structure's stability. This parameter has recently been used to study the energetic stability of other interesting nanostructured systems, such as nanoribbons and nanosheets.^87,88^ Note that the *E*_coh_ becomes less negative as the elements move from Al to In; this means that the (5,5) *ch*-AlPNT structure is the most stable, as predicted by the descriptor η. For instance, the cohesive energy of the bulk AlP and graphene-like AlP-structures are −8.41 and −7.63 eV/atom, respectively, whereas the corresponding value for (5,5) *ch*-AlPNT is >−4 eV/atom.^11^ The GaP nanocrystals and graphene-like GaP layers display *E*_coh_ < −13.4 and −9.25 eV/atom, respectively.^89,90^ The (5,5) *ch*-GaPNT shows *E*_coh_ > −3.7 eV/atom. InP crystals have cohesive energy of ∼−6.7 eV/atom, and the (5,5) *ch*-InPNT structures exhibit a value of >−3.5 eV/atom.^91^ Experimentally, the shape of the nanotubes tips or caps is largely governed by the synthesis method (laser ablation, the arc-discharge method, chemical vapor deposition, etc.), as well as the conditions and chemical species involved.^92−97^ On the other hand, open-ended nanotubes with hydrogen atoms attached to the dangling bonds (H-passivated) are a resource used for the finite molecular computational study of these nanostructures; however, as far as we are concerned, there are no experimental studies where this particular ending is emphasized. The results in Table 5 show that the *E*_coh_ values for *cap-hole* nanotubes are even lower compared to nanotubes passivated with hydrogen atoms. This suggests that in a possible competing reaction between these two types of terminations (*ch* or *-H*), the formation of *ch*-XPNTs would be favored. The cohesive energy for the XPNT-H was calculated with eq 11, which is equivalent to eq 8, where *E*_H_ is the total energy of the free hydrogen atom and *c* is the number of H atoms per nanotube.
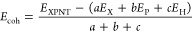
11

**Table 5:** Cohesive Energies (eV/Atom) for the (5,5) *ch*-XPNT (*ch* Column) and (5,5) XPNT-H (*-H* Column) Structures as Calculated with the LanL2DZ Basis Set and the M06-2X, PBE, and B3LYP Functionals

***E*_coh_**	(5,5) AlPNT		(5,5) GaPNT		(5,5) InPNT	
	*ch*	*-H*	*ch*	*-H*	*ch*	*-H*
M06-2X	–3.9402	–3.7224	–3.6959	–3.5133	–3.4680	–3.3006
PBE	–4.0743	–3.8196	–3.8774	–3.6511	–3.6191	–3.4138
B3LYP	–3.6719	–3.5067	–3.4494	–3.3142	–3.2171	–3.0985

**Figure 6:**
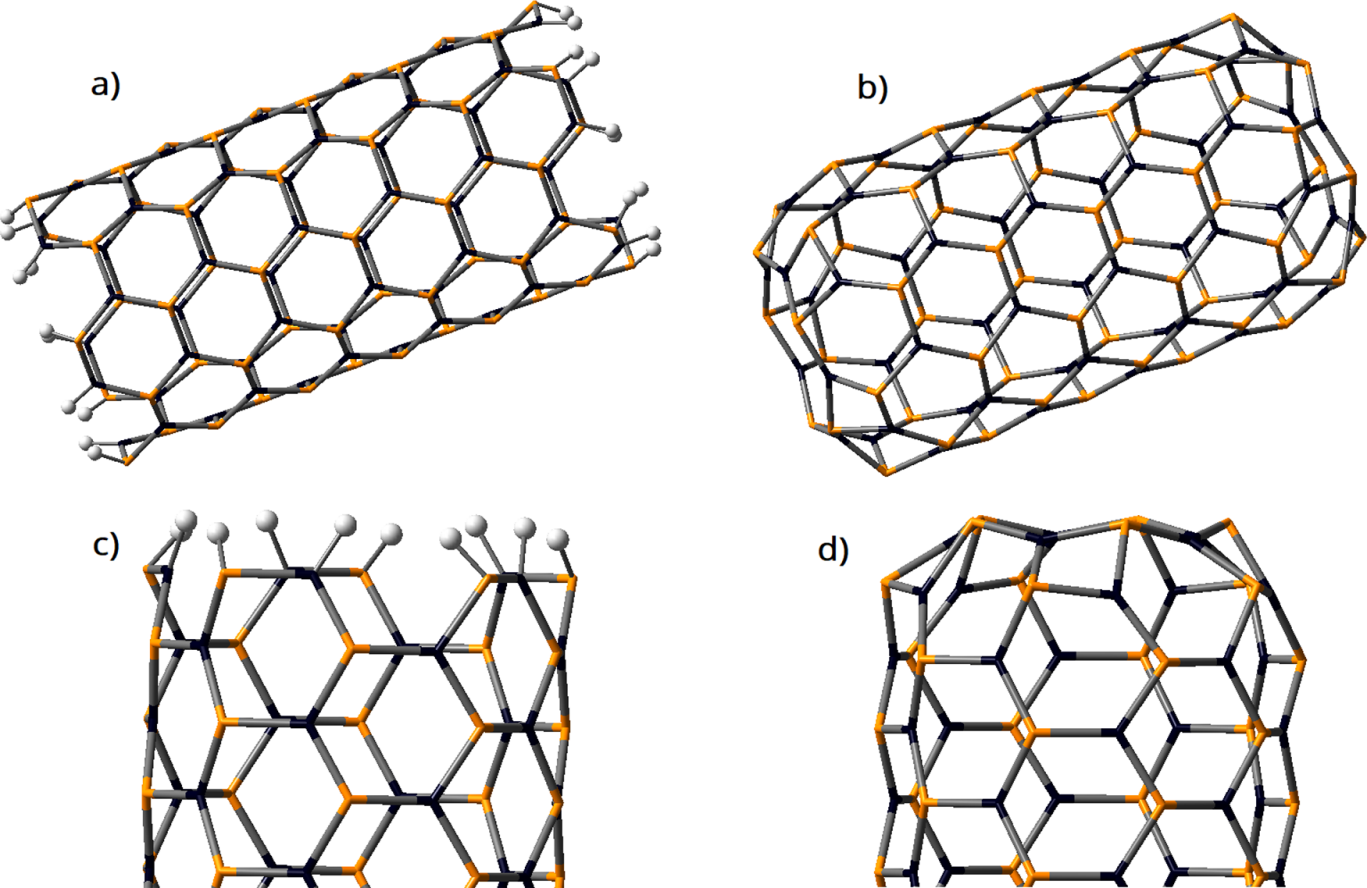
Nanotube model of (a) (5,5) XPNT-H and (b) (5,5) *ch*-XPNT, (c) close-up of the open end that is saturated with hydrogen atoms, and (d) close-up of the cap-hole structure proposed in this work. The black vertices correspond to the metal X atoms (X = Al, Ga, In), whereas the orange vertices represent the P atoms. Hydrogen atoms are represented as white balls.

The (5,5) XPNT-H is made up of 60 atoms of X (X = Al, Ga, In), 60 P atoms, and 20 H atoms, for a fair comparison with (5,5) ch-XPNTs (Figure 6).

#### 3.3.3 Dipole Moment (μ→), Polarizability (α), and Solvation Energy (Δ*E*_solv_)

Table S1 shows the dipole moment, polarizability, and solvation energy of all investigated NTs. It is well-known that the *open-end* armchair NTs display null polarity as a consequence of the structural symmetry, regardless of the atomic species of which they are made.^60,80,83,98,99^ The μ*→* values close to zero indicate that the curvature at the end of the new armchair cap-hole nanotubes induces only small distortions to the structure in such a way that they remain nonpolar systems. The polarizability (α) increases as one changes atoms from Al to In. Recall that α measures the molecule response capability to an electric field; consequently, it increases if the hardness is low. Results show a correlation between polarizability and energy gap: the (5,5) *ch*-InPNT is the system with the lowest *E*_g_ values and at the same time the largest polarizability. It is also possible to establish a relationship between the polarizability and the molecular energy gap: small *E*_g_ values (low global hardness system) correspond to a more polarizable molecule.

On the other hand, all solvation energy (Δ*E*_solv_) values, as calculated with eq 9 and summarized in Table S1, are negative, which mean a spontaneous and thermodynamically favorable solvation process. The Δ*E*_solv_ values calculated with the M06-2X and B3LYP functionals are quite similar. The magnitude of the solvation energies increases with the metal atomic number of block *p*, (5,5) *ch*-InPNT are the most stable.

**Figure 7:**
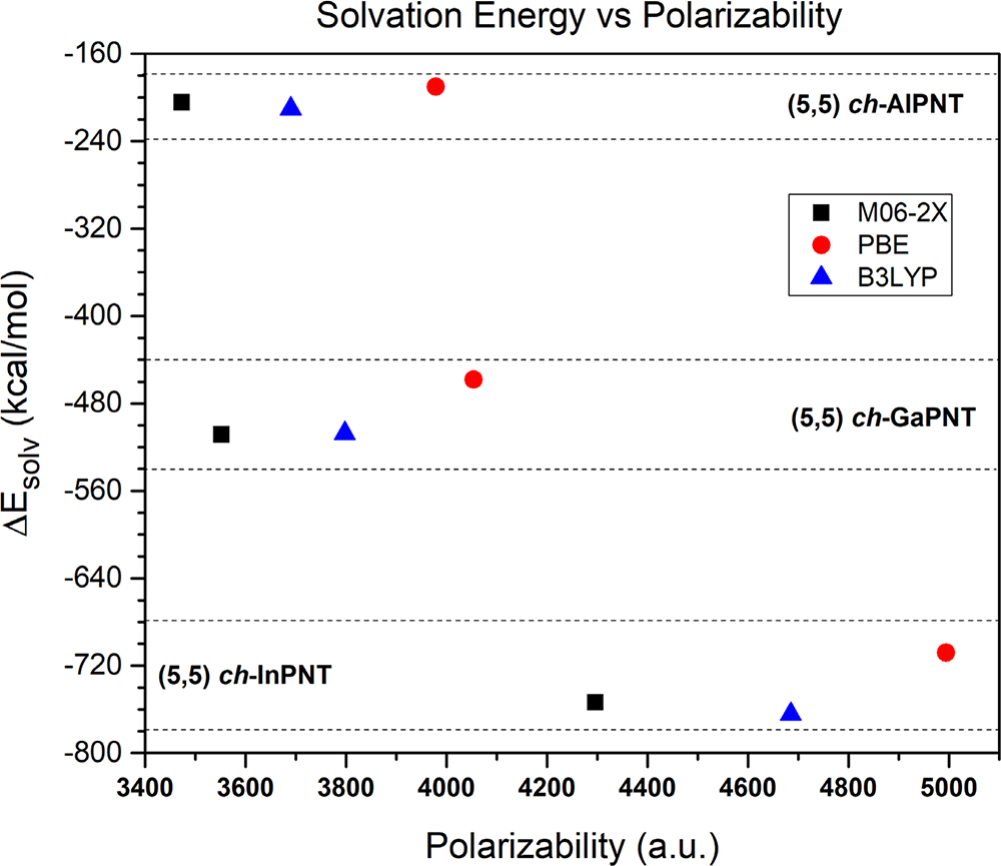
Solvation energy Δ*E*_solv_ (kcal/mol) vs polarizability α (a.u.). These were calculated with the functionals M06-2X (black line, squares), PBE (red line, circles), and B3LYP (blue line, triangles).

The intermolecular forces are key to the solvation processes. These predominant forces in a system depend on the solute polarity. Taking into account these facts, it is noted that the (5,5) *ch*-XPNT structures behave as nonpolar systems. These suggest that the (5,5) *ch*-XPNT solvation in water may take place through a mechanism different from that of the dipolar. One possible way may be through induced dipoles. Therefore, in this way, the NTs displaying large polarizability may form easily induced dipoles. This is corroborated in Figure 7, which shows the relationship between the Δ*E*_solv_ and the polarizability (α): as the polarizability increases, the solvation energy magnitude also increases; this is the most favorable process.

## 4 Conclusions

The inorganic *cap-hole* nanotubes' (in the armchair chirality) structural, vibrational, and electronic properties have been investigated, considering the (5,5) *ch*-XPNT configurations, where X = Al, Ga, and In. Studies have been done by performing first-principle calculations within the density functional theory and using the M06-2X, PBE, and B3LYP functionals, together with the LanL2DZ set basis, as well as charge neutrality and nonmagnetic calculations. The relaxed structures with constrained ends are formed as a consequence of the 10-atom rings arranged at the end of the nanotubes, with the absence of any passivation. These 10-atom ring edges have alternating cycles of six and four species that induce distortions in the X–P bond lengths. Note that all X–P's exhibit a single bond nature. The vibrational frequency results show how the nanotubes reach the global energy minima. The global molecular descriptors and the cohesive energy values of the (5,5) *ch*-AlPNT and (5,5) *ch*-InPNT structures correspond to the most and least electronically stable, respectively. The |HOMO–LUMO| energy gap (*E*_g_) increases with the atomic number in the *p* block element, where the PBE functional underestimates the energy gap (1.3 < *E*_g_ < 1.7 *eV*), whereas the M06-2X functional overestimates this energy gap (*E*_g_ > 3.5 *eV*). Under the B3LYP functional calculations, the NTs exhibit a semiconductor character in 2.1 < *E*_g_ < 2.8 eV. The large ω values of the (5,5) *ch*-XPNT structures indicate the electrophilicity of the NTs. Finally, the Δ*E*_solv_ negative values correspond to NTs with favorable solvation process, in particular, in the (5,5) *ch*-InPNT. The null dipole moment in the (5,5) *ch*-XPNT (μ*→* ≈ 0) and the relationship between the solvation energy Δ*E*_solv_ and polarizability α suggest that solvation is dominated by intermolecular forces produced by the induced dipoles instead of the permanent dipoles. These results have encouraged us to delve into future research about the formation and growth mechanisms (atom migration phenomenon) of these new nanotubes, as well as their impact on the stability of the system, as has been recently studied for SiCNTs and CNTs.^100,101^ The new inorganic NTs studied here are interesting structures that deserve attention not only from the scientific community but also from engineers for possible technological applications in sensor devices and drug transportation. Based on the modes, frequency relation, and optical absorption displayed by the systems at hand, we suggest applications of these NTs in optoelectronics, photonics, and biotechnology. Also, because of the electronic properties where the energy gap is found in the range 2.1 < *E*_g_ < 2.8 eV, we propose that these structures could be applied in the fabrication of integrated circuits at the nanoscale. Finally, because of the presence of metals (Al, Ga, and In), we may also suggest these NTs for application in spintronics.

## Software and Hardware Details

All calculations were performed with the software Gaussian 16,^36^ Revision C.01, by means of two processors (Intel Xeon E5-2680v3 and 30 M cache, 2.50 GHz, and 24 cores with a total RAM of 512 GB). Optimization was performed via DFT/M06-2X/LanL2DZ, DFT/PBE/LanL2DZ, and DFT/B3LYP/LanL2DZ, providing a seven-digit precision.

## Supporting Information Available

Bar graph of the HOMO–LUMO gap; (Figure S2) behavior of the molecular gap energy and chemical potential as function of the functional employed; (Table S1) values of the dipole moment, solvation energy, and polarizability of all nanotubes; and (supplementary equations) development of the equations that relate global hardness to molecular gap energy (PDF)
