# Supplementary material for: New Cap-Holed AlP, GaP, and InP Nanotubes
Source: ACS Omega. 2024 Jan 2;9(2):2920–30. doi: 10.1021/acsomega.3c08486 (PMC10795121; doi:10.1021/acsomega.3c08486)
Supplement: Supplementary file 1 — ao3c08486_si_001.pdf [file ao3c08486_si_001.pdf]

Supporting Information

New Cap-Holed AlP, GaP and InP Nanotubes

Raúl Mendoza-Báez [†], *Dolores García-Toral* [††,*], *Juan Francisco Rivas-Silva* [†††], *Akari Narayama Sosa Camposeco* [††††], *Sandra Esteban Gómez* [††††], *Gregorio Hernández Cocoletzi* [††††] *and Antonio Flores-Riveros* [††††]

[†]Departamento de Química, Centro de Investigación y de Estudios Avanzados del IPN (Cinvestav), Av. IPN 2508, Col. San Pedro Zacatenco, México City 07360, México.

[††]Benemérita Universidad Autónoma de Puebla, Facultad de Ingeniería Química, Av. San Claudio y 18 Sur S/N, San Manuel, Puebla 72570, México.

[†††] Meritorious Autonomous University of Puebla, Instituto de Física, Mexico.

[††††]Instituto de Física, Benemérita Universidad Autónoma de Puebla, Av. San Claudio y Blvd. 18 Sur, Col. San Manuel, Puebla 72570, México.

[*]*Correspondence author: dolores@ifuap.buap.mx*

3.3 Electronic Properties

3.3.1 HOMO and LUMO Orbitals

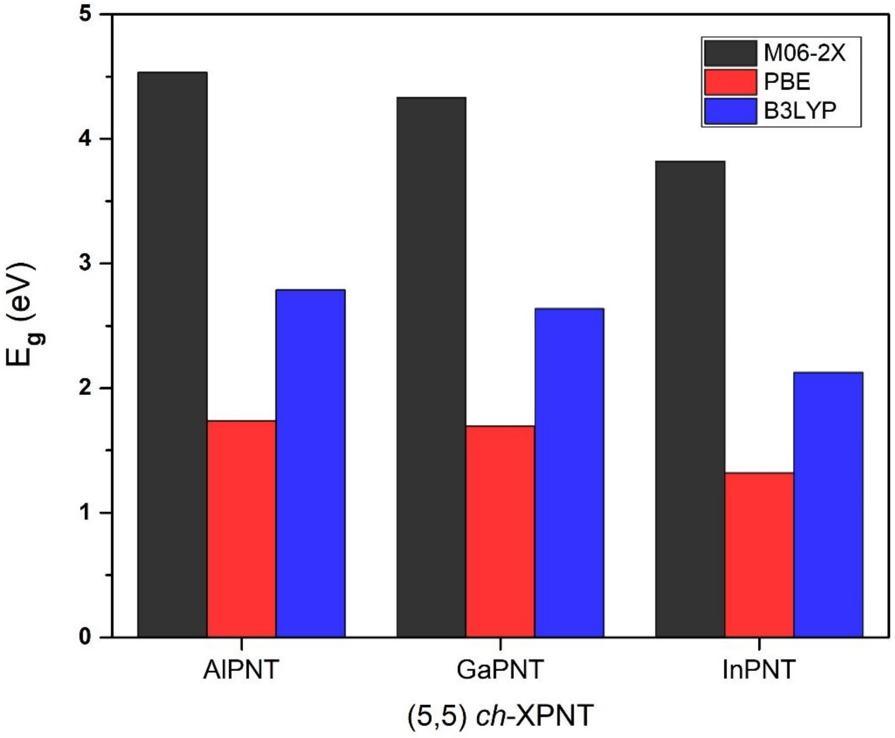

Figure S1. Bar graph of the energy gap |HOMO-LUMO| of the (5,5) *ch*-XPNT nanotubes as function of the functionals: M06-2X (black bar), PBE (red bar) y B3LYP (blue bar).

3.3.2 Global Molecular Descriptors and the cohesive energy E_{coh}

The global hardness (η) accounts for the system resistance to the charge transference, therefore, a better stability will have high η values. It is noted that the η values decrease as the group III elements go from Al to In, that is, the (5,5) *ch*-AlPNT and (5,5) *ch*-InPNT structures, which in turn exhibit the most and least electronic stability, respectively. Also, it is the similar trend observed in the molecular gap E_g.

This is because these two parameters are directly proportional, as shown in the development of the following equations:

$$E_g = |E_{HOMO} - E_{LUMO}| = |-I + A|$$

$$\eta = \frac{I - A}{2} \;\therefore\; -2\eta = -I + A$$

$$E_g = |-2\eta| = 2|\eta|$$

$$\eta = \frac{E_g}{2}$$

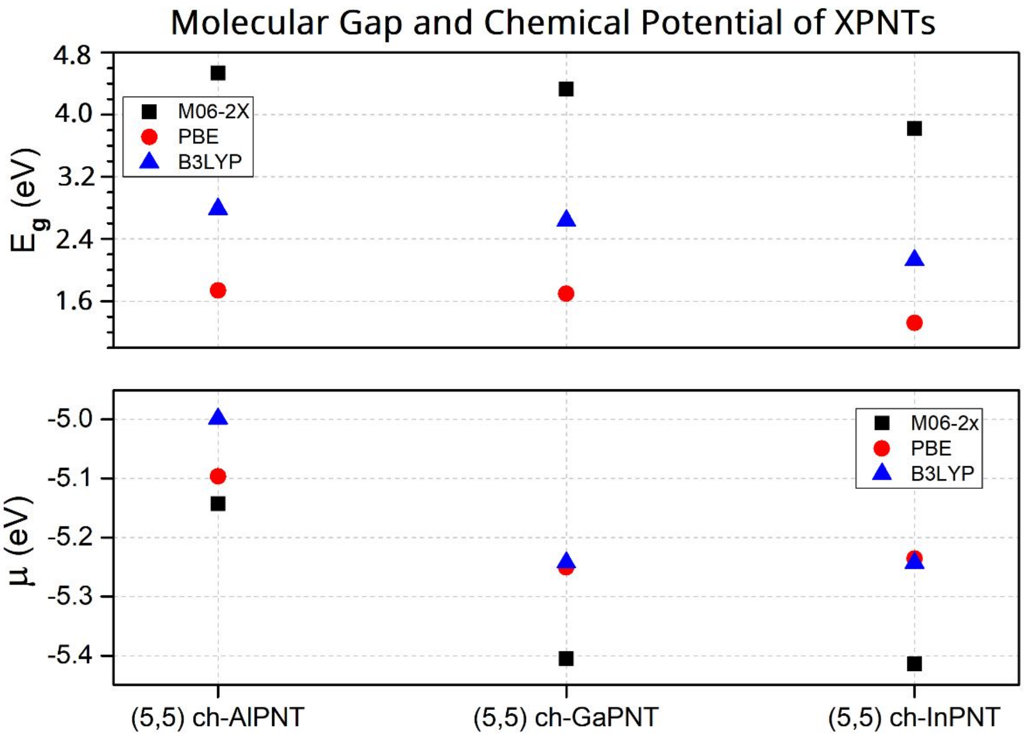

Figure S2. General behavior of the energy (eV) corresponding to the molecular gap (E_g) (up) and chemical potential (μ) (down) of all (5,5) *ch*-XPNT structures as function of the functionals employed in the calculations: M06-2X (black line, squares), PBE (red line, circles) y B3LYP (blue line, triangles).

3.3.3 Dipole moment ($\vec{\mu}$), polarizability (α) and solvation energy (ΔE_{solv})

In polyatomic systems, the dipole moment ($\vec{\mu}$) is the sum of all bond dipoles, which in turn allows distinguishing between polar and non-polar molecules. The Table S1 shows the dipole moments of all investigated NTs, which are non-polar, provided that all $\vec{\mu}$ are quite small, close to zero. It is well known that the armchair NTs display null polarity as a consequence of the structural symmetry, and at the same time this is independent on the NT chemical nature.

Table S1. Dipole moment $\vec{\mu}$ (Debye), solvation energy ΔE_{solv} (kcal/mol) and polarizability α (a.u.) of the (5,5) *ch*-XPNT (X = Al, Ga, In) structures.

**Nanotube**	**Dipole Moment (Debye)**	**E_solv (kcal/mol)**	**Polarizability (a. u.)**
**(5,5) *ch*-AlPNT**			
M06-2X	0.000028	-204.60	3472.76
PBE	0.000226	-190.19	3978.62
B3LYP	0.000003	-210.31	3690.27
**(5,5) *ch*-GaPNT**			
M06-2X	0.026423	-508.41	3552.35
PBE	0.000117	-458.26	4053.81
B3LYP	0.000199	-507.30	3797.62
**(5,5) *ch*-InPNT**			
M06-2X	0.000056	-753.42	4296.17
PBE	0.000001	-708.05	4994.00
B3LYP	0.000069	-764.26	4685.17
